# Health-related quality of life assessment in trials testing tyrosine kinase inhibitors or immune checkpoints inhibitors in early-stage NSCLC

**DOI:** 10.1093/oncolo/oyaf339

**Published:** 2025-10-07

**Authors:** Fabio Salomone, Giorgia Novero, Oriana Ciani, Roberto Ferrara, Alberto Servetto, Narjust Florez, Massimo Di Maio, Gabriella Pravettoni, Cecilia Pompili

**Affiliations:** Department of Clinical Medicine and Surgery, University of Naples Federico II, Naples, Italy; Department of Oncology, University of Turin, Turin, Italy; Bocconi School of Management, Center for Research on Health and Social Care Management, Milan, Italy; Department of Medical Oncology, IRCCS Ospedale San Raffaele, Milan, Italy; Università Vita-Salute San Raffaele, Milan, Italy; Department of Clinical Medicine and Surgery, University of Naples Federico II, Naples, Italy; Dana-Farber Cancer Institute, Harvard Medical School, Boston, MA, United States; Department of Oncology, University of Turin, Turin, Italy; Department of Oncology and Hemato-Oncology, University of Milan, Milan, Italy; Applied Research Division for Cognitive and Psychological Science, IEO, European Institute of Oncology IRCCS, Milan, Italy; Department of Thoracic Surgery, Institute for Clinical & Applied Health Research, University of Hull, Hull, UK

**Keywords:** early-stage, NSCLC, lung cancer, quality of life, HRQoL, perioperative, neoadjuvant, adjuvant

## Abstract

**Background:**

Health-related quality of life (HRQoL) remains underassessed and underreporting in randomized clinical trials (RCTs) evaluating new therapies in metastatic non-small cell lung cancer (NSCLC). However, evaluation and preservation of favorable HRQoL are critically important in trials including patients in early-stage settings, in which the primary objective is cure. Herein, we evaluated whether HRQoL was adequately evaluated and reported in trials including immune checkpoint inhibitors (ICIs) and tyrosine kinase inhibitors (TKIs) in resectable NSCLC.

**Methods:**

A systematic search was performed on Embase and PubMed to identify RCTs testing TKIs or ICIs in resectable NSCLC. We selected full articles and abstracts from major meetings. Risk of bias and reporting assessment of HRQoL were collected.

**Results:**

As of October 2024, we identified 25 RCTs. The primary endpoint was overall survival for 2 RCTs, while 21 and 7 RCTs evaluated risk of recurrence and tumour response as (co)-primary endpoints, respectively. Twelve RCTs (48%) did not assess HRQoL as an endpoint, while 13 (52%) included HRQoL evaluation as a secondary or exploratory endpoint. The most common tools utilized were FACT-L (6/13; 46%), EORTC-QLQ30/LC13 (4/13; 30%) and SF-36 (2/13; 15%). Phase II (33%) and adjuvant (44%) trials evaluated HRQoL in a lower rate than phase III (62%) and neoadjuvant/perioperative (66%) RCTs. Three out of 22 RCTs (14%) with available full-texts reported HRQoL results in the primary publication. Two out of the 19 remaining RCTs reported HRQoL in an indipendent publications, and 2 of them presented data in meeting abstracts. Remarkably, for 15 (68%) RCTs HRQoL evaluation is not available.

**Conclusions:**

Our systematic evaluation revealed suboptimal evaluation and underreporting of HRQoL in patients treated with novel agents and combinations in resectable NSCLC. Systematic evaluation and reporting of HRQoL should be prioritized in future trials.

Implications for Practice Our findings highlight the critical need for systematic assessment and transparent reporting of health-related quality of life (HRQoL) in resectable NSCLC trials. Given that cure and long-term well-being are primary goals in early-stage disease, incorporating HRQoL endpoints can guide clinicians in selecting therapies that balance efficacy with patients’ quality of life. By prioritizing HRQoL in clinical research, healthcare professionals can better understand the real-world impact of treatments, improve shared decision-making, and implement patient-centered care strategies in routine clinical practice.

## Background

The increasing adoption of lung cancer screening programs and the growing awareness about the disease are partially shifting the stage at diagnosis from metastatic (which remains the most common presentation) to potentially curable and early-stage lung cancer.^1^ With the aim of increasing the cure rates in a historically fatal disease, many clinical trials investigating novel treatments have been recently conducted focusing on early-stage non-small cell lung cancer (NSCLC), increasing curing rates to a level never seen before.^2^ However, their lives will still be affected by the physical and psychological consequences of the tumor and treatments received.^3^^,^^4^ As the efficacy of the clinical treatments, both pharmacological and non-pharmacological, has progressively increased, clinicians and patients are now interested not only in traditionally reported treatment outcomes and adverse events but also in how these treatments are affecting the patients’ health-related quality of life (HRQoL), both during the treatment phase and long term.^5^ Furthermore, global regulatory agencies are interested to systematically evaluate patients’ perspectives and needs during the process of drug development, approval and acceptability (ie, Patient-Focused Drug Development for Food and Drug Administration).^6^ In this vein, including preferences in treatment options of patients with lung cancer and their needs in medical decision related to cancer journey could be relevant to the care process.^7^^,^^8^ In addition to their role as endpoints within clinical trials, HRQoL tools have also been incorporated by the European Society for Medical Oncology—Magnitude of Clinical Benefit Scale (ESMO-MCBS) group to assess the overall benefit of novel strategies across different treatment populations and settings.^9^ Moreover, patients’ self-reported health status plays a prognostic role, especially in early-stage NSCLC. Indeed, the physical component of HRQoL is associated with both overall and cancer-specific survival in patients undergoing surgery for early-stage NSCLC.^10^ A standardized collection of HRQoL data in clinical trials will complement other prognostic factors in presurgical decision-making and allow for true informed consent for our patients.

HRQoL is measured through validated tools that provide information on aspects of their health status that are relevant to their quality of life, including symptoms, functionality, and physical, mental, and social health.^11–13^ They are used to assess a patient’s health status at a particular point in time, during the entire course of treatment, and/or after its conclusion.^11^ HRQoL evaluations are increasingly used both in patients’ routine care and in clinical research, where self-reported measures are important to compare treatments.^14^ However, despite the availability of 17 different validated tools specifically designed for patients with lung cancer,^15^ HRQoL assessments in clinical trials testing novel treatment in NSCLC are still underreported.^16^ In the perioperative setting, evaluating the impact of multi-modality treatment will contribute to more refined risk stratification, offering HRQoL questionnaires as integrative measures that capture multiple prognostic dimensions.^17^

As for clinical practice, currently, HRQoL measurements in patients with lung cancer are not yet part of the standard of care in most centres. Indeed, as for other cancers, like breast and prostate cancer, patients affected by lung cancer are not systematically offered the opportunity by their physicians to express the impact of lung cancer diagnostics and treatments on their HRQoL (including symptoms, functionality, and physical, mental, and social health).^18^ Most importantly, in many cases, these discussions are triggered by patients or caregivers themselves when their daily life is already significantly affected by their cancer treatments. Furthermore, symptoms like dyspnoea may reflect a subjective experience not fully captured by objective tests. Instead, self-reported physical functioning will be more sensitive to limitations than assessment reported by clinicians. Collecting results from HRQoL measures will play a key role in identifying physically and emotionally vulnerable patients who could benefit from targeted preoperative interventions, such as pre-rehabilitation with exercise training or additional psychological support, ultimately enhancing recovery trajectories.^19^ Gathering, developing and standardising HRQoL information for lung cancer during trials and everyday care can help patients make better informed decisions after diagnosis of life-impacting and life limiting conditions.^20^

This is a comprehensive, expert scientific review of the literature, identified by systematic searches about the adoption of HRQoL as endpoints in clinical trials conducted in lung cancer, with a specific focus on clinical trials conducted with immune checkpoint inhibitors (ICIs) and/or tyrosine kinase inhibitors (TKI) in neoadjuvant/adjuvant/perioperative setting.

## Methods

Our systematic review was conducted in accordance with the statement from Preferred Reporting Items for Systematic Reviews and Meta Analysis (PRISMA) guidelines.^21^ The full protocol was registered to the PROSPERO online platform (CRD42025649558).

A systematic literature search was performed using PubMed-indexed journals and the EMBASE database. Articles published up to October 2024 were included in the initial review. The research strategies included the terms linked to the lung cancer (“lung cancer” OR “NSCLC”) and the treatment strategies (“neoadjuvant” OR “adjuvant” OR “perioperative”). Studies meeting all the following conditions were considered eligible for further analysis: (1) phase II or phase III randomized clinical trials (RCTs) enrolling patients with early-stage NSCLC; (2) trials testing ICIs and/or TKI-based regimen in adjuvant/neoadjuvant/perioperative setting; (3) only studies within the English literature were included. Trials were excluded from our analysis if they investigated other concomitant local treatments, as radiation treatment, as well as retrospective trials, trials testing non-pharmacological therapies, trials in unresectable locally advanced and metastatic settings, phase I trials, trials of supportive care, brief reports, and studies testing only chemotherapy-based regimen. List of exclusion and inclusion criteria are summarized in Table S1. We selected both full-text publications and relevant abstracts or posters presented at major scientific conferences. Information was retrieved from all available sources, including main and secondary publications, supplementary materials, full study protocols, abstract, and posters.

The primary objective of this systematic review was to assess whether and how HRQoL outcomes were reported in RCTs testing ICIs and/or TKIs in early-stage NSCLC. The secondary objective was the rate of reported HRQoL assessment in trials, which include its analysis, type of questionnaire administered for HRQoL evaluation, type of publication in which HRQoL data were reported, and risk of bias assessment.

Two authors (F.S. and G.N.) independently evaluated the eligibility criteria of all screened articles using titles, abstracts, full texts, and study protocols. Any disagreements concerning the eligibility of studies were resolved by the third researcher (C.P.) through group discussion and full-text review. For each eligible trial, the following data were extracted and recorded into an electronic database: trial acronym, type of treatment regimen, phase of the trial, use of placebo-controlled design, class of investigational drug, primary endpoint, presence of HRQoL evaluation, questionnaire(s) used for HRQoL evaluation, timing of evaluation, and reporting of HRQoL data. Similarly, the possible occurrence of bias was evaluated by three authors (F.S, G.N, and C.P) according to Rob2 tool, and the discrepancies were resolved by consensus.^22^

## Results

### Study characteristics

Up to October 2024, literature search identified 1539 records after duplication removal (364 records) (Figure S1). One thousand four hundred forty-three records did not meet the inclusion criteria, a total of 25 phase II and III RCTs evaluating ICIs and/or TKI–based regimens in early-stage NSCLC were identified. The complete list of trials is presented in Table S2. The main characteristics of the RCTs included in our analysis are summarized in Table 1. Among the selected RCTs, only 2 out of 25 (8%) RCTs adopted overall survival (OS) as the primary endpoint. Specifically, 15 trials (60%) used disease-free survival (DFS), 6 trials (24%) used event-free survival (EFS), 2 (8%) major pathological response (MPR), 1 (4%) objective response rate (ORR) and 4 (16%) pathological complete response (pCR) as their primary endpoints. Regarding trials design, 9 RCTs (36%) were double blind while 16 (64%) were open label. As for the study phase, 9 trials (36%) were phase II RCTs while 16 trials (64%) were phase III. In addition, 16 trials (64%) tested an experimental drug in the adjuvant phase, while 3 (12%) and 6 (24%) trials were in the neoadjuvant and perioperative design, respectively. In 10 RCTs (40%) ICIs were tested with or without chemotherapy, while 15 trials (60%) tested TKI. Overall, 9 RCTs (36%) provided multimodality treatment, including surgery, in the trial protocol, while 16 trials (64%) included only systemic treatment. Twenty RCTs (80%) met their primary endpoint.

**Table 1.** Characteristics of the phase II and III RCTs included in the analysis.

	n	%
**Whole Series**	25	100%
**Primary endpoint** ^a^		
**DFS**	15	60%
**EFS**	6	24%
**MPR**	2	8%
**ORR**	1	4%
**OS**	2	8%
**pCR**	4	16%
**Masking**		
**Blinded**	9	36%
**Open label**	16	64%
**Study design**		
**Phase II**	9	36%
**Phase III**	16	64%
**Treatment setting**		
**Adjuvant**	16	64%
**Neoadjuvant**	3	12%
**Perioperative**	6	24%
**Type of experimental therapy**		
**Immunotherapy**	2	8%
**Immunotherapy plus chemotherapy**	8	32%
**Targeted therapy**	15	60%
**Surgical assessment** ^b^		
**Performed**	9	36%
**Not performed**	16	64%
**Study results**		
**Primary endpoint achieved**	20	80%
**Primary endpoint not achieved**	5	20%

a Endpoints are not mutually exclusive.

b Trial with surgical assessment potentially included multimodal treatment with surgery during trial protocol.

Abbreviations: DFS, disease free survival; EFS, event free survival; MPR, major pathological response; ORR, objective response rate; OS, overall survival; pCR, pathological complete response.

### Assessment of HRQoL as an endpoint

**Figure 1.**
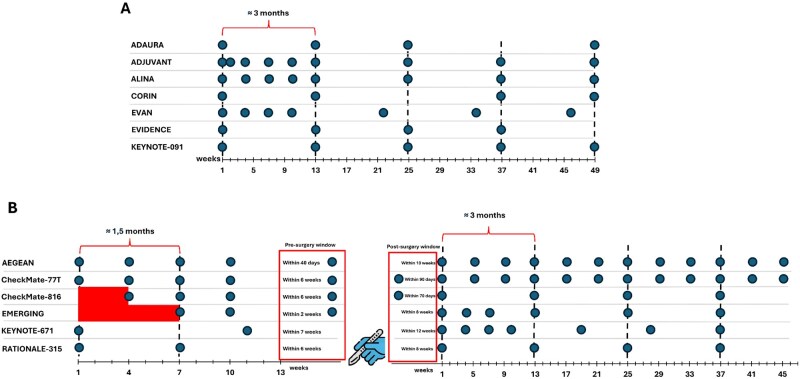
Timeline of HRQoL questionnaires administration in adjuvant RCTs (A) and in neoadjuvant/perioperative RCTs (B).

None of the 25 trials included HRQoL evaluation as a primary endpoint. HRQoL was evaluated as a secondary endpoint in 8 (32%) RCTs, while it was listed among exploratory endpoints in 4 (16%) trials. In 1 (4%) trial, HRQoL was included as both secondary and exploratory endpoints. Overall, 13 trials (52%) included HRQoL assessment as endpoint, while in the remaining 12 (48%) RCTs, HRQoL was not listed among endpoints at all. Notably, trials not evaluating HRQoL were predominantly open-label (56%), phase II (66%) and tested experimental treatment in the adjuvant setting (56%). Finally, 8 trials with positive results did not evaluate HRQoL among endpoints in study protocol. Further details regarding the inclusion of HRQoL assessment among trial endpoints based on study characteristics are reported in Table 2. All timeline descriptions of HRQoL questionnaire administration are reported in Figure 1.

**Table 2.** Inclusion of HRQoL among end points, based on study characteristics.

	Number of articles	HRQoL primary end point n (%)	HRQoL secondary end point n (%)	HRQoL secondary AND exploratory end point n (%)	HRQoL exploratory end point n (%)	HRQoL not evaluated as end point n (%)
**Whole series**	25	0	8 (32%)	1 (4%)	4 (16%)	12 (48%)
**Primary endpoint** ^a^						
**DFS**	15	-	5 (33%)	0 (0%)	2 (13%)	8 (53%)
**EFS**	6	-	2 (33%)	1 (16%)	2 (33%)	1 (16%)
**MPR**	2	-	1 (50%)	0 (0%)	0 (0%)	1 (50%)
**ORR**	1	-	1 (100%)	0 (0%)	0 (0%)	0 (0%)
**OS**	2	-	1 (50%)	0 (0%)	0 (0%)	1 (50%)
**pCR**	4	-	0 (0%)	1 (25%)	1 (25%)	2 (50%)
**Masking**						
**Blinded**	9	-	3 (33%)	1 (11%)	2 (22%)	3 (33%)
**Open label**	16	-	5 (31%)	0 (0%)	2 (12%)	9 (56%)
**Study design**						
**Phase II**	9	-	3 (33%)	0 (0%)	0 (0%)	6 (66%)
**Phase III**	16	-	5 (31%)	1 (6%)	4 (25%)	6 (38%)
**Treatment setting**						
**Adjuvant**	16	-	5 (31%)	0 (0%)	2 (12%)	9 (56%)
**Neoadjuvant**	3	-	1 (33%)	0 (0%)	1 (33%)	1 (33%)
**Perioperative**	6	-	2 (33%)	1 (16%)	1 (16%)	2 (33%)
**Type of experimental therapy**						
**Immunotherapy**	2	-	0 (0%)	0 (0%)	1 (50%)	1 (50%)
**Immunotherapy plus chemotherapy**	8	-	2 (25%)	1 (13%)	2 (25%)	3 (37%)
**Targeted therapy**	15	-	6 (40%)	0 ()	1 (7%)	8 (53%)
**Surgical assessment** ^b^						
**Performed**	9	-	3 (33%)	1 (11%)	2 (22%)	3 (33%)
**Not performed**	16	-	5 (31%)	0 (0%)	2 (12%)	9 (56%)
**Results of the trial**						
**Primary endpoint achieved**	20	-	7 (35%)	1 (5%)	4 (20%)	8 (40%)
**Primary endpoint not achieved**	5	-	1 (20%)	0 (0%)	0 (0%)	4 (80%)

a Categories are not mutually exclusive.

b “Surgical Assessment” included trials involving multimodal treatment with surgery as part of the protocol.

Abbreviations: DFS, disease free survival; EFS, event free survival; HRQoL, health-related quality of life; MPR, major pathological response; ORR, objective response rate; OS, overall survival; pCR, pathological complete response.

### HRQoL data reporting

Among the 22 trials with an available primary publication, we found that only 3/22 (14%) RCTs reported HRQoL data in the primary publication (Table 3). The absence of HRQoL data in primary publication was consistent across different trial characteristics (Table 3). Specifically, none of the phase II trials (100%) and 11 out of 14 phase III trials (79%) reported HRQoL in the primary publication. In addition, when analyzed by treatment setting, 86% (n = 12/14) of adjuvant trials and 87.5% (n = 7/8) of neoadjuvant/perioperative trials did not report HRQoL in their primary publication. Among the remaining 19 trials, only 2 RCTs (10%) disclosed HRQoL data in secondary publication and an additional 2 trials (10%) presented HRQoL findings in conference abstracts (Table S3). All HRQoL data results obtained from RCTs are summarized in Table 4.

**Table 3.** Disclosure of HRQoL results in primary publications, based on study characteristics.

	Number of articles	HRQoL results reported in primary publication n (%)	HRQoL results non-reported in primary publication n (%)
Whole series	22	3 (14%)	19 (86%)
Primary endpoint^a^			
DFS	13	2 (15%)	11 (84%)
EFS	5	1 (20%)	4 (80%)
MPR	1	0 (0%)	1 (100%)
ORR	1	0 (0%)	1 (100%)
OS	2	0 (0%)	2 (100%)
pCR	4	0 (0%)	4 (100%)
Masking			
Blinded	8	1 (12%)	7 (88%)
Open label	14	2 (14%)	12 (86%)
Study design			
Phase II	8	0 (0%)	8 (100%)
Phase III	14	3 (21%)	11 (79%)
Treatment setting			
Adjuvant	14	2 (14%)	12 (86%)
Neoadjuvant	3	0 (0%)	3 (100%)
Perioperative	5	1 (20%)	4 (80%)
Type of experimental therapy			
Immunotherapy	2	0 (0%)	2 (100%)
Immunotherapy plus chemotherapy	7	1 (14%)	6 (86%)
Targeted therapy	13	2 (15%)	11 (86%)
Surgical assessment			
Performed	8	1 (12%)	7 (88%)
Not performed	14	2 (14%)	12 (86%)
Results of the trial			
Primary endpoint achieved	17	3 (17%)	14 (83%)
Primary endpoint not achieved	5	0 (0%)	5 (100%)

a Endpoints are not mutually exclusive.

Abbreviations: DFS, disease free survival; EFS, event free survival; HRQoL, health-related quality of life; MPR, major pathological response; ORR, objective response rate; OS, overall survival; pCR, pathological complete response.

**Table 4.** HRQoL data reported in selected RCTs.

Clinical Trial	HRQoL Tool	Type of statistical analysis	Principal PROs Outcome	Functioning	Symptoms
**ADAURA**	SF-36	Norm-based scoring relative to the 2009 US general population. Within-group MIDs in mean change from baseline.	**PCS:** No baseline differences No longitudinal differences in terms of MMRM and TTD **MCS:** No baseline differences No longitudinal differences in terms of MMRM and TTD	Role Limitations–Physical, Social Function, Role Limitations- Emotional—Baseline greater impairment No baseline differences between arms in each domain No longitudinal differences between arms in each domain	N/A
**ADJUVANT**	FACT-L LCSS TOI	TTD Longitudinal differences with odds ratio using generalized estimated equations (GEE)	**FACT-L** Statistical difference at week 3 in Gefitinib arm, compared to control arm. TTD improved in the Gefitinib arm (HR 0.62, 95 % CI 0.42-0.90, *P* = 0.013). **LCSS** No statistical difference between arms at each timepoint. TTD increased in the Gefitinib arm (HR 0.63, 95% CI 0.43-0.93, *P* = 0.020). **TOI** No statistical difference between arms at each timepoint. Time to deterioration increased in the Gefitinib arm (HR 0.51, 95% CI 0.33-0.77, *P* = 0.001).	N/A	N/A
**ALINA**	SF-36	Norm-based scoring relative to the 2009 US general population. Within-group MIDs in mean change from baseline.	**PCS:** Mean change exceeds MIDs in the alectinib arm from baseline to week 96. Mean change exceeds MIDs in the chemotherapy arm from baseline to week 96. **MCS:** Mean change exceeds MIDs in alectinib arm from baseline to week 12 and 96. Mean change exceeds MIDs in the chemotherapy arm from baseline to week 96.	Alectinib arm improved **Bodily Pain**, **Role Physical**, **Mental health**, Social functioning, Vitality from baseline to week 12 Control arm declined General health and Vitality from baseline to week 12	N/A
**CheckMate77T**	FACT-L NSCLC-SAQ	TTD in disease-related symptoms MMRM	**NSCLC-SAQ:** Statistically significant difference in favour of experimental arm with median TTD of 40.0 months (95% CI, 33.6 to not reached) in the nivolumab group and a median of 31.1 months (95% CI, 25.0 to not reached) in the chemotherapy group (hazard ratio, 0.66; 95% CI, 0.45 to 0.98). no clinically meaningful change from baseline.	N/A	N/A
**CheckMate816**	EQ-5D-3L	MMRM	**VAS:** No differences from baseline to post neoadjuvant in each arm. No differences between arms. **UI:** No differences from baseline to post neoadjuvant in each arm. No differences between arms.	N/A	N/A
**EVIDENCE**	FACT-L LCSS	MMRM	**FACT-L:** No differences between arms from baseline to week 36. **LCSS:** No differences between arms from baseline to week 36.	N/A	N/A
**KEYNOTE-671**	EORTC QLQ-C30 EORTC QLQ-LC13	MMRM	**GHS:** Deterioration from baseline to neoadjuvant week 11 in both arms, but no differences between arms (1.43 (−1.64, 4.49)). Deterioration from baseline to adjuvant week 10 in the control arm, but no differences between arms (2.22 (−0.58, 5.02)).	**Physical Functioning (PF):** Deterioration from baseline to neoadjuvant week 11 in both arms, but no differences between arms (0.35 (−1.99, 2.68)) Deterioration from baseline to adjuvant week 10 in both arms, but no differences between arms (0.75 (−1.63, 3.13)) **Role Functioning (RF):** Deterioration from baseline to neoadjuvant week 11 in both arms, but no differences between arms (1.04 (−2.41, 4.49)) Deterioration from baseline to adjuvant week 10 in both arms, but no differences between arms (2.14 (−1.33, 5.61))	**Dyspnoea:** Deterioration from baseline to neoadjuvant week 11 in the control arm, but no differences between arms (−1.77 (−5.10, 1.55)) Deterioration from baseline to adjuvant week 10 in both arms, with differences in favour of experimental arm (-3.86 (−7.63, −0.09)) **QLC-LC13 Cough:** Improvement from baseline to neoadjuvant week 11 in both arms, but no differences between arms (−1.82 (−5.01, 1.36)) Improvement from baseline to adjuvant week 10 in both arms, but no differences between arms (−2.15 (−5.95, 1.65)) **Chest Pain:** No differences from baseline to neoadjuvant week 11 in both arms and no differences between arms (0.72 (−2.03, 3.47)) Deterioration from baseline to adjuvant week 10 in both arms, but no differences between arms (−3.04 (−6.53, 0.45))
**RATIONALE-315**	EORTC QLQ-C30 EORTC QLQ-LC13	MMRM TTD	**GHS:** No differences between arms	**Physical Functioning (PF):** No differences between arms	**Fatigue:** No differences between arms **Coughing:** Least Square improvement in favour of experimental arm (−4.37 [95% CI: −9.46 to 0.21]). **Chest Pain:** TTD in favour of experimental arm (HR 0.59 [95% CI: 0.38−0.91]) **Dyspnoea:** No differences between arms

Abbreviations: GHS, global health status; HRQoL, health-related quality of life; MCS, mental component summary; MIDs, minimal important differences; MMRM, mixed model of repeated measures; PCS, physical component summary; TTD, time to deterioration; UI, Utility Index; VAS Visual Analogue Scale.

### Risk of bias assessment

By selecting only RCTs, the risk of selection was reduced, as all RCTs were at low risk of selection bias, as the random sequence generation method was adequately reported, and low risk of deviations from the intended interventions. High risk of missing outcome data and measurement of the outcome was identified in two and four RCTs, respectively (Table S4). Some concerns appeared in the evaluation of the selection of the reported results in two trials.

## Discussion

Trials testing novel treatment in early stage failed to report adequate HRQoL measures, particularly phase II trials and many of the results from the HRQoL tools utilized were not included in the primary publication or submitted as independent abstracts, making the findings a secondary endpoint and decreasing the attention of the scientific community towards these results.

In detail, 52% of RCTs did not include HRQoL among the endpoints while 48% enlisted HRQoL evaluation among secondary or exploratory endpoints. In addition, assessment of HRQoL was reported in primary publication in 14% of RCTs with an available publication. Instead, 10% and 10% of RCTs reported HRQoL results in a secondary publication or in an abstract meeting, respectively. Finally, HRQoL results differed based on the type of questionnaires utilized and results emerging from RCTs. Altogether, these data revealed that HRQoL is still underreported and underestimated also in trials investigating treatment in early setting with no trial assessing HRQoL as the primary endpoint.

Recently, many regulatory agencies, ie, the European Medicines Agency (EMA) and Food and Drug Administration (FDA), recommended the incorporation of HRQoL evaluation in the development of novel anticancer strategies, shifting patients’ perception of disease and treatment in the middle of effectiveness assessment.^23^^,^^24^ In addition, the incorporation of ICIs in the neoadjuvant/adjuvant setting, as well as the use of TKIs in the adjuvant setting, has reshaped the treatment landscape of early-stage NSCLC, potentially increasing the cure rate for patients.^25^ In this context, evaluating patients’ perspectives is fundamental for selecting the most appropriate treatment, taking into account both the short-term consequences of multimodal approaches (surgery, systemic therapy, and radiotherapy) and their long-term effects.

Previous analyses conducted by Marandino and colleagues highlighted a suboptimal proportion of phase III trials evaluating HRQoL as a study endpoint, in oncology randomized phase III trials.^14^ Although the authors reported an increase in the rate of RCTs including HRQoL assessment, rising from 52.9% during 2012-2016 to 67.8% in the period 2017-2021, this improvement remains suboptimal.^14^^,^^26^ Further analysis from our group focusing on phase III trials evaluating systemic treatment for advanced NSCLC found that 23.3% of RCTs published between 2010 and 2021 did not assess HRQoL.^16^ Overall, our findings revealed that up to 48% of the trials identified in this systemic review omitted HRQoL evaluation in early-stage NSCLC settings regardless of treatment combinations ICIs vs TKIs. While survival remains the primary endpoint in the drug approval process, novel strategies, particularly in early-stage cancer settings, often require a prolonged follow-up period to understand long-term effects of therapies in the curative setting.^27^ As a result, surrogate endpoints such as pCR, MPR, or EFS are commonly used for drug approval.^28^ However, these treatments may be associated with long-term and permanent adverse effects, also years after beginning of treatment, that can significantly impact patients’ daily functioning and overall well-being without a potential benefit in overall survival and without an evaluation of patients’ perspective.^29^ Furthermore, trials that included HRQoL evaluation in their protocols showed significant variability in the timing of assessments and the duration of the follow up, largely due to differences in the instruments used.^30^ Firstly, the choice of a specific instrument may yield insights that could be missed if a different tool were used, like the lack of physical health symptoms evaluation in SF-36 questionnaire.^31^ Secondly, the predefined timepoints for HRQoL evaluation in RCTs often fail to capture key events such as disease recurrence, discontinuation of active therapy, and, more broadly, the impact of additional treatments like surgery.^24^ Therefore, a standardized approach is urgently needed, not only in terms of instruments, but also regarding the timing of HRQoL assessments.

The timeline of HRQoL tool administration highlights a lack of assessment and reporting immediately before and/or after surgery. Notably, 17% to 24% of patients enrolled in RCTs investigating neoadjuvant or perioperative chemoimmunotherapy did not proceed to surgery.^32–35^ Although disease progression is the primary reason for surgery cancellation, many patients also withdraw consent or decline to continue with the adjuvant phase. Collecting consistent, HRQoL data around the time of surgery could provide valuable insights into factors affecting adherence to treatment. While various metrics have been developed to classify the severity of surgery-related complications, their use as endpoints in perioperative trials remains limited.^36^ On the other hand, long-term assessment of patients’ quality of life after the completion of therapy is generally conducted at progressively wider intervals (every 3-6 months), which may limit the ability to accurately capture the patient’s lived experience and long-term perspective. Paratore and colleagues reported that up to 30% of RCTs conducted in advanced settings fail to report HRQoL outcomes.^37^ This finding raises concerns about the completeness of data available to guide patient-centered decision-making in oncology. In line with these observations, our own analysis showed that only one out of four trials with an available publication reported HRQoL outcomes, either in a primary or secondary publication. This limited reporting, at least in part, be explained by the recent publication dates of many of the included trials. In fact, previous research in the context of advanced NSCLC demonstrated that the likelihood of HRQoL data being reported in a secondary publication tends to increase 24 to 36 months after the initial publication of trial results.^16^ Therefore, it is possible that some of the trials in our cohort may still contribute HRQoL data soon. Nevertheless, the current underreporting emphasizes the need for more systematic and timely dissemination of HRQoL outcomes, which are crucial for evaluating the overall benefit of cancer therapies, particularly in the early disease settings where quality of life is a key component of care. It is important to take into consideration that HRQoL is a multidimensional concept that encompasses different areas of patient’s life, including not only physical aspects, but also individual’s psychological factors.^38^ Other aspects of a patients journey should be included in the HRQoL assessment including: emotions, personal perceptions and needs along the oncological pathway.^39^ Even partial HRQoL results can provide valuable insights, especially in the context of multimodal treatment approaches, where surgery is combined with systemic therapies. Nonetheless, to avoid an incomplete assessment that may result in premature conclusions, the definition within the protocol of the timing for questionnaire evaluation, combined with a more rigorous definition of the expected endpoints related to HRQoL assessment, can facilitate a more accurate interpretation of the obtained results. In addition, while the safety of a treatment is defined as the risk of developing clinical signs or abnormal laboratory tests related to its use, how well patients tolerate it may be overlooked.^40^ In these cases, assessing not only the risk of perioperative complications but also the patient’s subjective experience of recovery becomes essential for a comprehensive evaluation of treatment impact and for supporting shared decision-making. Promoting a shared decision making could be essential in order to increase patients’ involvement during oncological treatments, potentially reducing negative emotions and enhancing HRQoL.^41^

We acknowledge several limitations in our work. First, we included only clinical trials investigating TKIs or ICIs in early-stage NSCLC, thereby excluding trials evaluating chemotherapeutic regimens and resulting in a relatively limited sample size. However, the introduction of these novel therapies has significantly changed the treatment landscape, raising the bar for curative strategies in early-stage disease. Moreover, our analysis focused solely on the methodological assessment of HRQoL reporting within these trials and does not provide information on the actual impact of treatments on specific HRQoL domains. Thus, no conclusions can be drawn regarding the efficacy of TKIs or ICIs in improving or impairing different aspects of patients’ daily lives. Furthermore, due to the rapid evolving nature of the drug development in early-stage NSCLC, we will have not included studies that were presented in the most recent international conferences. Finally, focusing on the English literature would have excluded trials conducted in the Asian continent who is currently evaluating their own combination compounds in early-stage at an accelerated pace.

In conclusion, although ICIs and TKIs have revolutionized the management of early-stage NSCLC, it remains unclear how these therapies affect patients’ everyday functioning and well-being. Future studies should prioritize the integration and transparent reporting of HRQoL outcomes to ensure that therapeutic advancements translate into meaningful benefits from the patient’s perspective.

## Supplementary Material

oyaf339_Supplementary_Data

## Data Availability

The data underlying this article are available in the article and in its online supplementary material. For any other request for data related to the manuscript, please contact the corresponding author.
