# Supplementary material for: Health-related quality of life assessment in trials testing tyrosine kinase inhibitors or immune checkpoints inhibitors in early-stage NSCLC
Source: Oncologist. 2025 Oct 7;30(10):oyaf339. doi: 10.1093/oncolo/oyaf339 (PMC12558747; doi:10.1093/oncolo/oyaf339)
Supplement: oyaf339_Supplementary_Data [file oyaf339_supplementary_data.docx]

**Supplementary Materials.**

**Supplementary Figure 1.** PRISMA diagram

**Supplementary Table 1.** List of inclusion and exclusions criteria. Abbreviations: ICI Immune checkpoint inhibitor; NSCLC non-small cell lung cancer; RCTs Randomized controlled trials; TKI tyrosine kinase inhibitor.

**Supplementary Table 2.** List of RCTs included in the analysis. Abbreviations: ChT: Chemotherapy; DFS: Disease Free Survival; EFS: Event Free Survival; HRQoL: Health-Related Quality of Life; ICIs: immune checkpoint inhibitors; MPR: Major Pathological Response; ORR: Objective Response Rate; OS: Overall Survival; pCR: pathological Complete Response; TT: target therapy.

**Supplementary Table 3.** Rate of secondary publications reporting HRQoL results.

**Supplementary Table 4.** Risk of bias summary for each included study reporting HRQoL results

**Supplementary Figure 1**

**Supplementary Table 1**

| Inclusion Criteria | Exclusion Criteria |
| --- | --- |
| - phase II or phase III RCTs  - early-stage NSCLC  - trials testing ICIs and/or TKI -based regimen in adjuvant/neoadjuvant/perioperative setting  - English language | - trial with concomitant local treatments, like radiation treatment  - retrospective trials  - trials testing non-pharmacological therapies  - trials in unresectable locally advanced and metastatic settings  - phase I trials  - trials of supportive care  - brief reports  - studies testing only chemotherapy-based regimen |

| **Name of Study** | **Strategy** | **Study Design** | **ICIs** | **ICIs + Cht** | **TT** | **Primary Endpoint** | **Masking** | **Surgical Assessment** | **HRQoL PRIMARY endpoint** | **HRQol SECONDARY endpoint** | **HRQol EXPLORATORY endpoint** | **Test** | **HRQoL in primary publication** | **HRQoL  in secondary publication** | **HRQoL only in Meeting Abstract** |
| --- | --- | --- | --- | --- | --- | --- | --- | --- | --- | --- | --- | --- | --- | --- | --- |
| ADAURA | Adjuvant | III | NO | NO | YES | DFS | Blinding | NO | NO | YES | NO | SF-36 | NO | YES | NO |
| ADJUVANT | Adjuvant | III | NO | NO | YES | DFS | Open Label | NO | NO | YES | NO | FACT-L + TOI;  LCSS | YES | YES | NO |
| AEGEAN | Perioperative | III | NO | YES | NO | EFS, pCR | Blinding | YES | NO | YES | YES | EORTC QLQ-LC13, C30  EQ-5D-5L | NO | NO | NO |
| ALINA | Adjuvant | III | NO | NO | YES | DFS | Open Label | NO | NO | NO | YES | SF-36, EQ-5D-5L | NO | NO | YES |
| BR.19 | Adjuvant | III | NO | NO | YES | OS | Blinding | NO | NO | NO | NO | NA | NO | NO | NO |
| CheckMate77T | Perioperative | III | NO | YES | NO | EFS | Blinding | YES | NO | NO | YES | FACT-L; NSCLC-SAQ; EQ-5D-5L | YES | NO | NO |
| CheckMate816 | Neoadjuvant | III | NO | YES | NO | EFS, pCR | Open Label | YES | NO | NO | YES | EQ-5D-3L | NO | NO | YES |
| CORIN | Adjuvant | II | NO | NO | YES | DFS | Open Label | NO | NO | YES | NO | FACT-L; LCSS | NO | NO | NO |
| EMERGING | Neoadjuvant | II | NO | NO | YES | ORR | Open Label | YES | NO | YES | NO | FACT-L; LCSS | NO | NO | NO |
| EVAN | Adjuvant | II | NO | NO | YES | DFS | Open Label | NO | NO | YES | NO | FACT-L; LCSS | NO | NO | NO |
| EVIDENCE | Adjuvant | III | NO | NO | YES | DFS | Open Label | NO | NO | YES | NO | FACT-L; LCSS | YES | NO | NO |
| ICOMPARE | Adjuvant | II | NO | NO | YES | DFS | Open Label | NO | NO | NO | NO | NA | NO | NO | NO |
| ICTAN | Adjuvant | III | NO | NO | YES | DFS | Open Label | NO | NO | NO | NO | NA | NO | NO | NO |
| IMPACT | Adjuvant | III | NO | NO | YES | DFS | Open Label | NO | NO | NO | NO | NA | NO | NO | NO |
| IMpower010 | Adjuvant | III | YES | NO | NO | DFS | Open Label | NO | NO | NO | NO | NA | NO | NO | NO |
| KEYNOTE091 | Adjuvant | III | YES | NO | NO | DFS | Blinding | NO | NO | NO | YES | EQ-5D; EORTC QLQC30; LC13 | NO | NO | NO |
| KEYNOTE671 | Perioperative | III | NO | YES | NO | EFS / OS | Blinding | YES | NO | YES | NO | EORTC QLQC30; LC13  EQ-5D-5L | NO | YES | NO |
| NADIMII | Perioperative | II | NO | YES | NO | PCR | Open Label | YES | NO | NO | NO | NA | NO | NO | NO |
| neoTORCH | Perioperative | III | NO | YES | NO | EFS / MPR | Blinding | YES | NO | NO | NO | NA | NO | NO | NO |
| RADIANT | Adjuvant | III | NO | NO | YES | DFS | Blinding | NO | NO | NO | NO | NA | NO | NO | NO |
| RATIONALE315 | Perioperative | III | NO | YES | NO | EFS / MPR | Blinding | YES | NO | YES | NO | EORTC QLQ-C30 / LC13 | NO | NO | YES |
| TD-FOREKNOW | Neoadjuvant | II | NO | YES | NO | PCR | Open Label | YES | NO | NO | NO | NA | NO | NO | NO |
| 10.1016/j.esmoop.2024.102694 | Adjuvant | II | NO | NO | YES | DFS | Open Label | NO | NA | NO | NA | NA | NO | NO | NO |
| 10.1245/s10434-014-3586-9 | Adjuvant | II | NO | NO | YES | DFS | Open Label | NO | NO | NO | NO | NA | NO | NO | NO |
| 10.1371/journal.pone.0140794 | Adjuvant | II | NO | NO | YES | DFS | Open Label | NO | NO | NO | NO | NA | NO | NO | NO |

**Supplementary Table 2**

|  | Number of articles | QoL results reported in secondary publication n (%) | QoL results presented in meeting abstract n (%) | QoL results non-reported in secondary publication n (%) |
| --- | --- | --- | --- | --- |
| Articles non-reporting QoL results in primary publication | 19 | 2 (10%) | 2 (10%) | 15 (80%) |

**Supplementary Table 3**

**
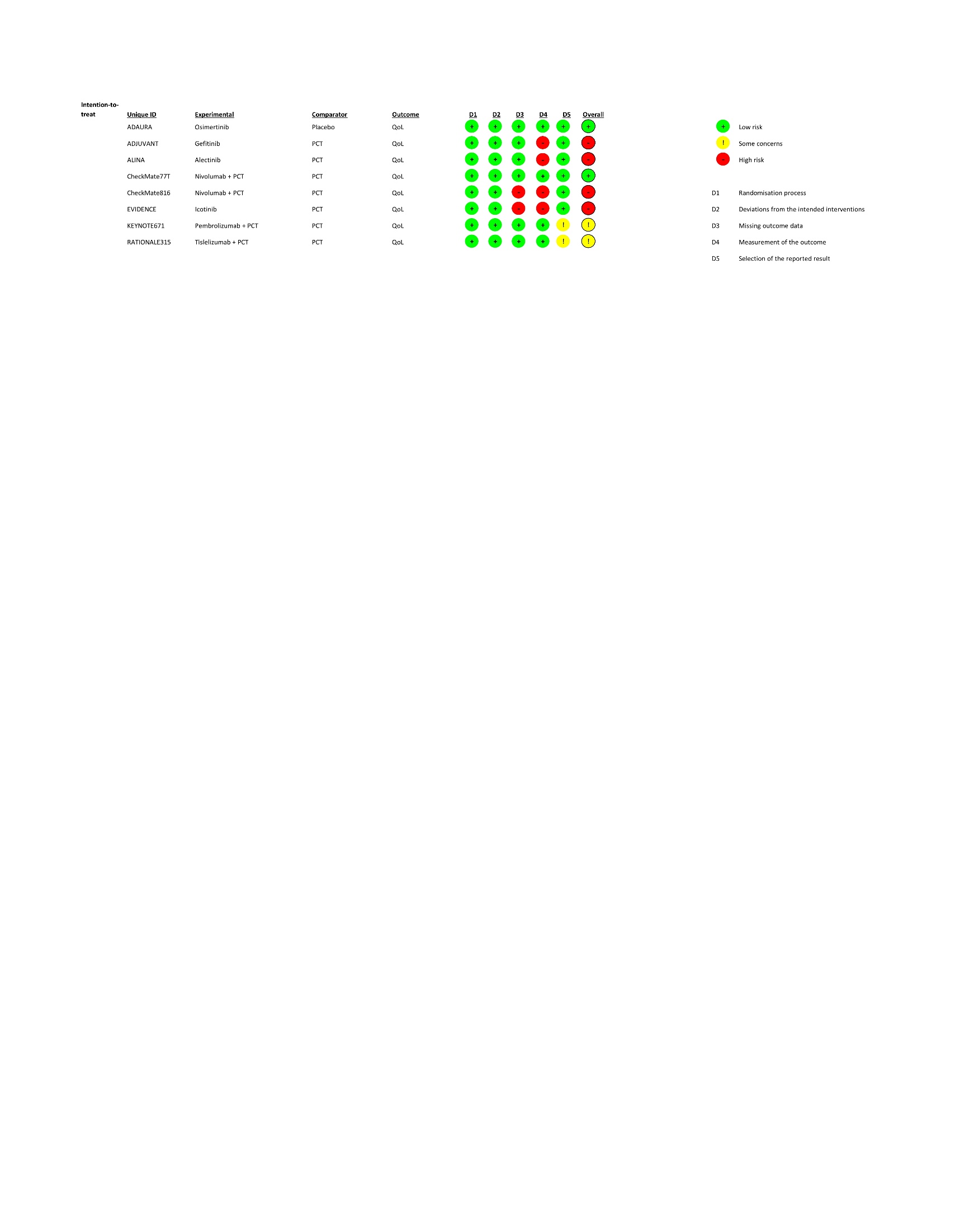
**

**Supplementary Table 4**
